# Toad Venom Antiproliferative Activities on Metastatic Melanoma: Bio-Guided Fractionation and Screening of the Compounds of Two Different Venoms

**DOI:** 10.3390/biology9080218

**Published:** 2020-08-10

**Authors:** Laura Soumoy, Mathilde Wells, Ahmad Najem, Mohammad Krayem, Ghanem Ghanem, Stéphanie Hambye, Sven Saussez, Bertrand Blankert, Fabrice Journe

**Affiliations:** 1 Laboratory of Human Anatomy and Experimental Oncology, Faculty of Medicine and Pharmacy, University of Mons, 7000 Mons, Belgium; laura.soumoy@umons.ac.be (L.S.); sven.saussez@umons.ac.be (S.S.); 2 Laboratory of Pharmaceutical Analysis, Faculty of Medicine and Pharmacy, University of Mons, 7000 Mons, Belgium; mathilde.wells@umons.ac.be (M.W.); stephanie.hambye@umons.ac.be (S.H.); bertrand.blankert@umons.ac.be (B.B.); 3 Laboratory of Oncology and Experimental Surgery, Institut Jules Bordet (ULB), 1000 Brussels, Belgium; ahmad.najem@bordet.be (A.N.); mohammad.krayem@bordet.be (M.K.); gghanem@ulb.ac.be (G.G.); 4 Department of Oto-Rhino-Laryngology, Université Libre de Bruxelles (ULB), CHU Saint-Pierre, 1000 Brussels, Belgium

**Keywords:** melanoma, targeted therapies, resistance to drugs, toad venom, cardiotonic steroids, sodium pump, marinobufagenin, bufalin, high performance liquid chromatography, mass spectrometry

## Abstract

Melanoma is the most common cancer in young adults, with a constantly increasing incidence. Metastatic melanoma is a very aggressive cancer with a 5-year survival rate of about 22−25%. This is, in most cases, due to a lack of therapies which are effective on the long term. Hence, it is crucial to find new therapeutic agents to increase patient survival. Toad venoms are a rich source of potentially pharmaceutically active compounds and studies have highlighted their possible effect on cancer cells. We focused on the venoms of two different toad species: *Bufo bufo* and *Rhinella marina*. We screened the venom crude extracts, the fractions from crude extracts and isolated biomolecules by studying their antiproliferative properties on melanoma cells aiming to determine the compound or the combination of compounds with the highest antiproliferative effect. Our results indicated strong antiproliferative capacities of toad venoms on melanoma cells. We found that these effects were mainly due to bufadienolides that are cardiotonic steroids potentially acting on the Na^+^/K^+^ ATPase pump which is overexpressed in melanoma. Finally, our results indicated that bufalin alone was the most interesting compound among the isolated bufadienolides because it had the highest antiproliferative activity on melanoma cells.

## 1. Introduction

Melanoma originates from melanocytes which derivate from pluripotent cells in the neural crest [1]. Melanoma is the deadliest form of skin cancer. It represents only 5% of all cases, but it is responsible for about 80% of skin cancer-related deaths [2,3]. Its incidence has been steadily increasing for more than 20 years and it represents the first form of cancer in young adults (25–29 years old) [4]. When diagnosed at an early stage, the tumor can easily be treated by surgery, but advanced stage melanomas are particularly difficult to treat. Indeed, the 5-year survival rate is about 22–25% for metastatic melanoma patients [4]. This poor survival rate is linked to a lack of effective therapies. Indeed, most melanoma cells are radio and chemo-resistant, mainly due to their melanin production which protects DNA. The current treatments for metastatic melanoma rely on immunotherapy and targeted therapies [5]. The main targeted therapies used in metastatic melanoma treatment act on the MAPK signaling pathway, which is mutated in BRAF and NRAS in about, respectively, 50% and 30% of patients with cutaneous melanoma [6,7]. Another important target for these types of therapy is the tyrosine kinase receptor cKIT which is mutated in about 20% of patients with mucosal melanoma [8]. Inhibition of the MAPK pathway shows initially good response, but the large majority of metastatic melanoma patients treated with targeted therapies develop resistances within weeks or months following the onset of treatment [9]. Indeed, the major challenge with such inhibitors is that melanoma cells possess an hypermutable genome and can activate many alternative signaling pathways, leading to acquired resistance to such therapies [5]. Regarding the lack of therapies with long-term efficacy in patients with stage IV melanoma who do not respond to immunotherapy, the need for new therapeutic candidates with anticancer properties represents an absolute necessity. The study of their effect on parental cells—as well as on cells with acquired resistance to current targeted therapies—is of great interest.

The wide diversity of biologically active compounds provided by our worldwide flora and fauna (plants, fungi, bacteria and animals) is a well-known statement and empirically exploited by our ancestors and used in traditional medicines. It has given rise to different scientific disciplines which study natural products and has highlighted numerous pharmacologically active substances, essentially in plants. Some of them were patented and approved by official authorities in our therapeutic arsenal [10]. In more recent years, animal venoms/secretions have been the source of an ever more growing scientific interest. Animals use their venoms mainly to trap their preys and to defend themselves. This represent a very wide range of venoms coming from many different species: spiders, scorpions, snakes, marine species, but also toads. This work focuses on toad venoms. These venoms have been extensively used for years in traditional medicine in Asia, South America and Egypt [11,12]. A well-known example is *HuaChanSu*, a traditional Chinese medicine composed of dried extracts of toad skin and used mainly for its anti-inflammatory, cardiotonic, local anesthetic and diuretic properties [13,14,15]. *HuaChanSu* is also used in traditional Chinese medicine for the treatment of certain types of cancer [16]. Toad venoms are a rich and varied source of pharmacologically active compounds. The main compounds that can be found are biogenic amines, bufadienolides, peptides and proteins and alkaloids [17,18]. These compounds are responsible for a large panel of potential therapeutic indications such as cardiotonic/anti-arrhythmic, antidiabetic, immunomodulatory, antibacterial/antifungal, antiprotozoal, antiviral, antineoplastic, sleep inducing, analgesic, contraceptive, endocrine activity, behavioral changes or wound healing [19,20]. Among antiproliferative activities, some specific ones have already been highlighted on melanoma cells [21,22,23].

In this work, we wanted to evaluate the antiproliferative effect of toad venoms and their components on melanoma cells. This project was initiated because studies have indicated that these venoms act on the Na^+^/K^+^ ATPase [14,15] which is overexpressed in many cancer types, including metastatic melanoma [24]. More interestingly so, a recent study indicated that bufalin, a molecular compound found in toad venom, was endogenously expressed in human serum and the concentrations were decreased in the case of hepatocellular carcinoma [25]. We used a bio-guided fractionation to isolate the different compounds found in toad venoms. We focused on two toad species: *Bufo bufo* and *Rhinella marina*. *Bufo bufo* is the common toad mainly found in Europe. *Rhinella marina*—better known as the cane toad—is predominantly present in Central and Latin America and some parts of Australia. These two toad species were selected because they have already shown strong anticancer effects on hematological, solid, sensitive and/or resistant human tumor cell lines [14,26,27,28]. Their compound fingerprints have already been widely studied and so has their anticancer effects on different cancer types. However, this is, to our knowledge, the first study investigating and comparing the effect of crude extracts, fractions and isolated compounds alone or in combination on melanoma cell lines either sensitive or resistant to targeted therapies.

## 2. Materials and Methods

### 2.1. Extraction of Toad Venom

The dried toad venom samples (*Bufo bufo*; *Rhinella marina*) were provided by Alphabiotoxine^®^ (Montrœul-au-bois, Belgium). The extraction process was performed following the procedure previously described by Lenaerts et al. [29]. Briefly, 100 mg of ground venom was submitted to a sonication-assisted extraction in 5 mL of methanol for 2 h at room temperature. The resulting extract was centrifuged for 30 min at 1920× *g*. The supernatant was collected and stored at −20 °C in a brown-glass vial. This process was applied for both the *Bufo bufo* and *Rhinella marina* venom. For the purpose of the crystal violet assay, the methanolic crude extracts were evaporated to dryness using a miVac^®^ centrifugal concentrator (Hyogo, Kansai, Japan). The dry residues were then solubilized in DMSO 100% (Sigma-Aldrich, MO, USA) to a concentration of 1.0 mg/mL. The samples were then diluted to the desired concentration.

### 2.2. Preparation of Standard Solutions

Reference standards of three authentic bufadienolides were purchased. Telocinobufagin was supplied by Musechem^®^ (New Jersey, NJ, USA). Bufalin and Resibufogenin were supplied by PhytoLab^®^ (Vestenbergsgreuth, Bavaria, Germany). One reference compound was isolated in-house (marinobufagenin) following the purification process described by Lenaerts et al. [29]. Briefly, this compound was isolated by a preparative thin-layer chromatography approach followed by a semipreparative LC isolation. Individual standard stock solutions were prepared by dissolving 1.0 mg in 1.0 mL of DMSO.

### 2.3. UHPLC Instrumentation and Analysis

The LC system was made up of a Waters^®^ Acquity UHPLC H-Class instrument (Waters^®^, Milford, CT, USA) and was equipped with a quaternary pump and a 2 × 48-vial autosampler. The separation was performed on an Acquity UPLC HSS T3 column (100 × 2.1 mm, i.d.; particle size: 1.8 µm) from Waters^®^. Column temperature was set to 40 °C and the thermostated autosampler was maintained at 10 °C. The instrument was equipped with a PDA and QDa detector allowing for MS detection (Waters^®^, Milford, CT, USA), equipped with an electrospray ionization source operating in positive ionization mode (ESI +) and a simple quadrupole. The cone voltage was set at 15 V. Control of the whole system and data acquisition were managed via Empower^®^ software (Waters, version 3.1). The mobile phases were composed of A = ultrapure water (18.2 MΩ × cm) with 0.1% formic acid and B = acetonitrile with 0.1% formic acid. Gradient elution was used according to the following method: 20% of mobile phase B from 0 to 0.5 min, gradually increased to 30% B at 2 min, going from 30% to 35% B in 4 min and from 35% to 60% B in 3 min and set at 95% B at 10 min, 20% B at 12 min and maintained isocratic until 14 min (for column re-equilibration). The injection volume was 10 μL and the mobile phase flow rate was set at 0.450 mL/min. UV detection wavelength was set at 296 nm. QDa full mass scan was allowed over a wide range of masses going from 100 to 1000 Da [29].

### 2.4. Cell Lines and Culture

Three cell lines harboring different mutations were selected: HBL (^D820Y^cKIT), MM074 (^V600E^BRAF) and MM161 (^Q61R^NRAS). These lines are sensitive to targeted therapies, but we also worked with their resistant counterparts. To develop these resistances, cells were chronically exposed to increasing doses of targeted therapies for 12 weeks (0.01 µM during Week 1 and 2; 0.05 µM during Week 3 and 4; 0.1 µM during Week 5 and 6; 0.5 µM during Week 7 and 8; 1 µM during Week 9 and 10; 2 µM during Week 11 and 12). This model mimics what happens in patients who develop resistances to targeted therapies. Both sensitive and resistant cell lines were provided by the Laboratory of Oncology and Experimental Surgery at Institut J. Bordet, Université libre de Bruxelles (Brussels, Belgium).

Cells were grown in Ham-F10 medium (Lonza, Bâle, Switzerland) supplemented with 10% fetal bovine serum and 1% penicillin/streptomycin (both from Life Technologies, Carlsbad, CA, USA) at 37 °C in a humidified 95% air and 5% CO_2_ atmosphere. The cultures were confirmed to be free of mycoplasma contamination using PCR-based detection.

### 2.5. Effectors

The BRAF inhibitor dabrafenib, used for BRAF mutated cells and the MEK inhibitor Pimasertib, used for NRAS mutated cells, were from Selleck Chemicals (Houston, TX, USA). The tyrosine kinase inhibitor dasatinib—used for cKIT mutated cells—was from Bristol-Myers (New-York, NY, USA).

### 2.6. Viability Assay

Cells were first seeded in a 96-well plate (10,000 cells/well for sensitive cells and 12,000 cells/well for resistant cells) and left to adhere for 24 h. DMSO 0.1% was used as negative control. New media containing the different extracts/fractions/molecules was then added in each well. The plates were then incubated for a further 72 h. Following the treatment period, cells were washed with DPBS (Life technologies, Carlsbad, CA, USA) and fixed with glutaraldehyde 1.5% for 15 min (Sigma-Aldrich, MO, USA). Cells were then stained with a 5% crystal violet solution for 30 min (Sigma-Aldrich, MO, USA), the staining dye was discarded, and cells were washed with distilled water. Cells were then incubated with Triton 0.2% (Rhom & Haas Co., PA, USA) for 90 min under constant agitation at room temperature so as to permeabilize cell walls. Absorbance was read at 570 nm using a spectrophotometer VERSAmax (Molecular Devices, Sunnyvale, CA, USA). For each condition, we performed 3 biologic replicates and 6 technical replicates for each biologic replicate.

### 2.7. qPCR Analysis

Total mRNA was extracted from cultured cells using Qiagen RNeasy Mini Kit (Qiagen, Venlo, Netherlands). Extracted mRNA was collected in RNAse free water and concentrations were evaluated using a BioDrop Duo (BioDrop, Cambridge, UK). Total RNA was treated with DNAse I Amplification Grade (Thermo Fisher Scientific, Waltham, MA, USA). cDNA was synthesized using Maxima First Strand cDNA Synthesis Kit (Thermo Fisher Scientific, Waltham, MA, USA). Real-time PCR reactions were performed using the FastStart essential DNA green master (Roche, Basel, Switzerland). Relative quantification was determined by normalizing the crossing threshold (CT) of ATP1A1 with the CT of 18S (loading control) using the method 2-ΔCT. Primer sequences for ATP1A1 are: Fwd: 5′- GGC CTT TAA GGT TGG ACG TG-3′, Rev: 5′- CAC AGT AAC ATT GAG AAC CCC C-3′.

### 2.8. Statistical Analysis

The IC_50_ values represent the inhibitory concentrations producing 50% growth reduction and were calculated from dose–response curves using GraphPad Prism software (GraphPad Software, La Jolla, CA, USA). All data concerning viability are expressed as means ± standard deviation. For each condition, 3 biologic replicates were performed, each composed of 6 technical replicates. Statistical significance was measured by Student’s t-test using GraphPad Prism software. All the statistical analyses are available in Supplementary material (Tables S1–S12). Comparison of mean IC50 values are conducted with ANOVA test and Tukey post hoc test using IBM SPSS Statistics 23 (IBM, Ehningen, Böblingen, Baden-Württemberg, Germany). In all analyses, *p* < 0.05 was considered to be statistically significant (marked with *) and *p* < 0.01 and *p* < 0.001 was considered highly statistically significant (marked, respectively with ** and ***).

## 3. Results

### 3.1. Antiproliferative Activity of Crude Extracts

**Figure 1:**
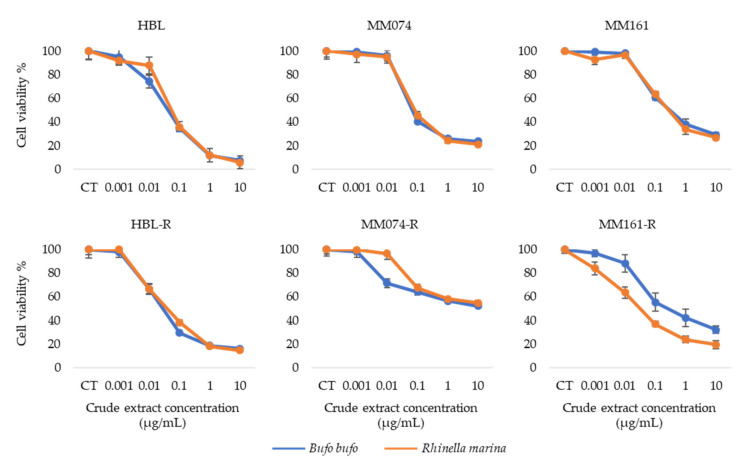
Graphs representing the percentage of viability (%) (*y* axis) at different crude extract concentrations (µg/mL) (*x* axis) in each cell line.

First, we performed crystal violet assays to study and compare the antiproliferative properties of *Bufo bufo* and *Rhinella marina* crude extracts (see chemical profiles in Figure S1) on our three parental sensitive melanoma cell lines and on their three resistant counterparts (Figure 1).

These results indicate a similar decrease in cell viability for both extracts in all cell lines excepted in the case of MM161-R which is NRAS mutated and has developed an acquired resistance to pimasertib. For this particular cell line, we can observe that the decrease in viability is greater when cells are treated with the crude extract from *Rhinella marina* rather than the extract from *Bufo bufo* venom, with an IC_50_ 8 times smaller for *Rhinella marina* (IC_50_ *Rhinella marina* = 0.05 µg/mL; IC_50_ *Bufo bufo* = 0.40 µg/mL).

### 3.2. UHPLC Analysis of Rhinella Marina Crude Extract Fractions

**Figure 2:**
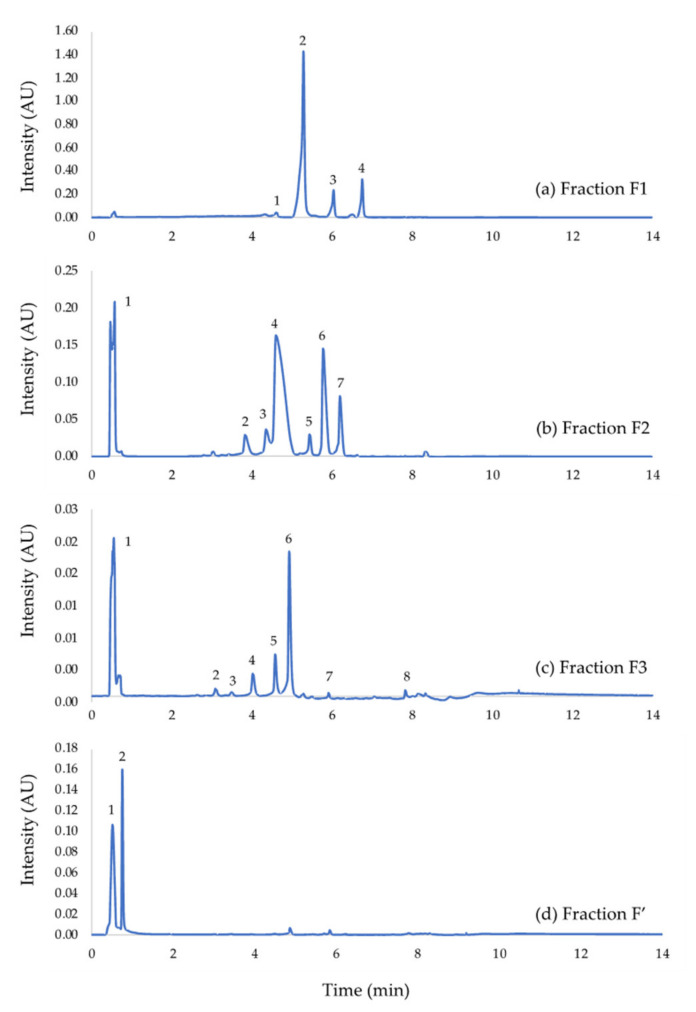
UHPLC-UV/MS analyses of: (**a**) Fraction F1—1: telocinobufagin ([M+H]^+^ = 403.4 *m/z*); 2: marinobufagenin ([M+H]^+^ = 401.4 *m/z*); 3: bufalin ([M+H]^+^ = 387.4 *m/z*); 4: Resibufogenin ([M+H]^+^ = 385.3 *m/z*); (**b**) fraction F2—1: suberoyl arginine ([M+H]^+^ = 331.2 *m/z*); 2: marinobufagin−3-pimeloyl-arginine ([M+H]^+^ = 699.7 *m/z*); 3: telocinobufotoxin ([M+H]^+^ = 715.6 *m/z*); 4: marinobufotoxin ([M+H]^+^ = 713.7 *m/z*); 5: marinobufagin−3-adipate-arginine ([M+H]^+^ = 685.7 *m/z*); 6: bufalitoxin ([M+H]^+^ = 699.7 *m/z*); 7: resibufotoxin ([M+H]^+^ = 697.7 *m/z*); (**c**) fraction F3—1: suberoyl arginine ([M+H]^+^ = 331.2 *m/z*); 2: marinobufagin−3-adipate-arginine ([M+H]^+^ = 685.6 *m/z*); 3: telocinobufagin−3-pimeloyl-arginine ([M+H]^+^ = 701.1 *m/z*); 4: marinobufagin-3-pimeloyl-arginine ([M+H]^+^ = 699.7 *m/z*); 5: telocinobufotoxin ([M+H]^+^ = 715.6 *m/z*); 6: marinobufotoxin ([M+H]^+^ = 713.7 *m/z*); 7: bufalitoxin ([M+H]^+^ = 699.7 *m/z*); 8: unknown identity; (**d**) fraction F’—1: dehydrobufotenin ([M+H]^+^ = 203.2 *m/z*).

The hereabove shown results indicate similar antiproliferative properties for both venoms, but a higher impact of exposure to *Rhinella marina* extract in MM161-R cells is highlighted. For this reason, the methanolic crude extract from *Rhinella marina* venom was studied further. The crude extract was fractionated into four fractions (fraction F1; fraction F2; fraction F3; fraction F’). The fractionation method cannot currently be detailed owing to confidentiality agreements. The chemical profile for each fraction was determined by LC–MS (Figure 2).

Initial peak identification was performed by comparing parent ion [M+H]^+^ mass to charge ratios to published detailed LC-QTOF and HPLC–ESI–MS/MS analyses of several *Rhinella marina (L.)* toad venom sample [27,30,31,32,33,34,35]. Fraction F1 mainly contains free hydrophobic bufagenins. Fraction F2 and F3 possess similar chemical profiles. They are principally made up of conjugated hydrophilic bufagenins, better known as bufotoxins. Biogenic amines are also present, but in a smaller proportion. Finally, the main compound found in fraction F’ is an indole alkaloid (Table 1).

### 3.3. Antiproliferative Activity of Rhinella Marina Venom Fractions

**Figure 3:**
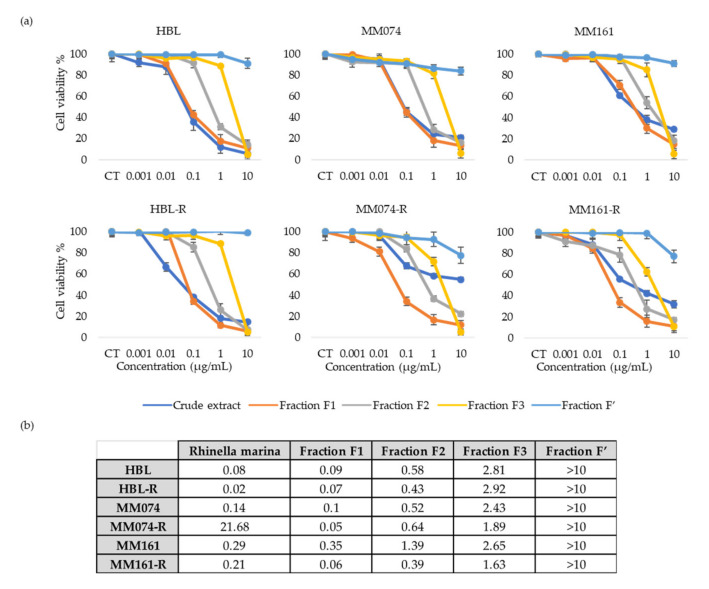
(**a**) Graphs representing the percentage of viability (%) (*y* axis) at different fraction concentrations (µg/mL) (*x* axis) in each cell line; (**b**) evaluation of the IC_50_ (µg/mL) of each crude extract (*Rhinella marina*) and fraction (F1, F2, F3, F’) in the 3 parental melanoma cell lines and their respective resistant ones.

We performed crystal violet assay to study and compare the antiproliferative properties of the 4 fractions obtained from *Rhinella marina* crude extracts on our three parental sensitive melanoma cell lines and on their three resistant counterparts (Figure 3).

These data indicate that the total extract and fraction F1 have a similar effect on the viability of the 3 parental sensitive cell lines and of the resistant HBL-R cell line. However, fraction F1 exerts a greater on MM074-R and MM161-R which both harbor mutations of the MAPK pathway. In addition, the viability curves obtained with fraction F1, which contains mostly bufadienolides, allow us to conclude that the effect is dose-dependent. Another interesting point is that, at the highest tested concentration (10 µg/mL), the percentage of residual viability (5–15%) is approximately the same for fractions F1, F2 and F3. However, for fractions F2 and F3, which contains mainly bufotoxins, the decrease in viability percentages begins at higher concentrations and is more abrupt. The IC_50_ values obtained when treating with F1 are about 3 to 12 times smaller than with F2 and 7 to 40 times smaller than with F3. Finally, for fraction F’, who’s main component in an alkaloid, displays very weak antiproliferative properties. In the higher ranges of concentration, we observed only 2–23% of viability inhibition.

### 3.4. Antiproliferative Activity of “Fraction F1” Compounds

**Figure 4:**
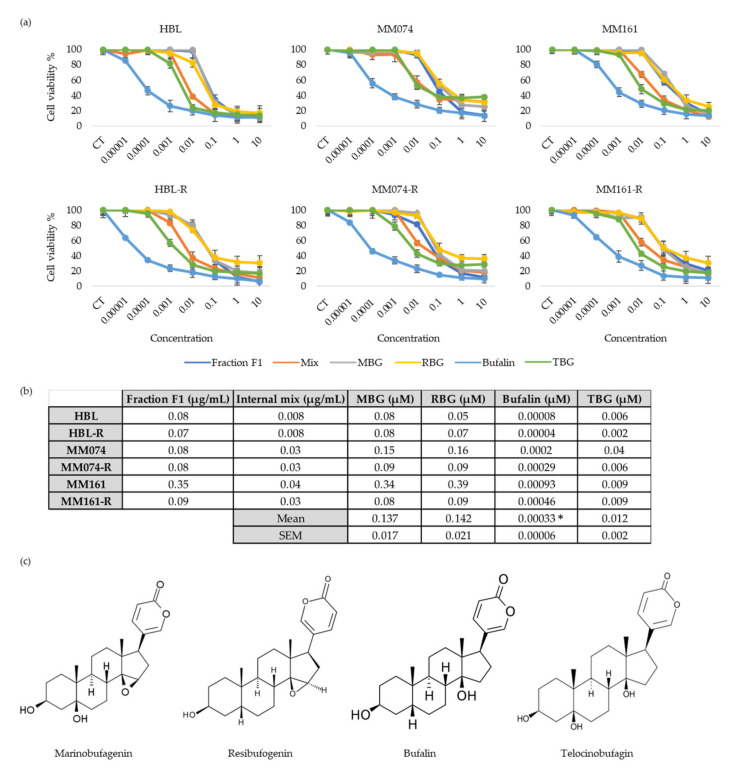
(**a**) Graphs representing the percentage of viability (%) (*y* axis) at different fraction, mix and bufadienolides concentrations (µg/mL for fraction F1 and internal mix; µM for MBG, RBG, bufalin and TBG) (*x* axis) in each cell line; (**b**) evaluation of the IC_50_ in the 6 cell lines; (**c**) chemical structures of marinobufagenin (MBG); resibufogenin (RBG); bufalin and telocinobufagin (TBG). * *p* < 0.05 vs MBG and RBG (ANOVA test and Tukey’s post hoc test)

Considering that fraction F1 gave rise to the most interesting IC_50_ values, the next step of our work was to evaluate and compare the antiproliferative activities of the main components of this fraction individually. Fraction F1 is constituted of marinobufagenin (MBG) (65.50%), resibufogenin (RBG) (10.27%), bufalin (8.88%) and telocinobufagin (TBG) (2.48%). Of note, this fraction also contains 12.87% of unidentified molecules. To check that these unidentified compounds were not responsible for the antiproliferative activity of fraction F1, we also prepared an in-house mix containing the four bufadienolides mentioned above with the same respective proportions for each compound (Figure 4).

The results indicate a similar dose–response pattern when the cells are treated either with fraction F1, the mix or individual molecules. The IC_50_ values obtained for the internal mix were lower than those obtained with the total fraction (F1). Indicating that the major antiproliferative properties observed when cells are exposed to fraction F1 are mainly linked to the activity of the four identified bufadienolides. MBG and RBG which both possess an epoxy group have similar antiproliferative activities, but their antiproliferative effect is relatively weak when compared to the effect of bufalin and TBG, two bufadienolides without an epoxy group. Bufalin is the bufadienolide with the higher antiproliferative properties. Its antiproliferative effect is already observed at the lowest tested concentration (0.01 nM) and thus in each cell line. IC_50_ s vary between 0.1 and 1.0 nM.

### 3.5. Antiproliferative Activity of Bufalin Combined with other Bufadienolides

**Figure 5:**
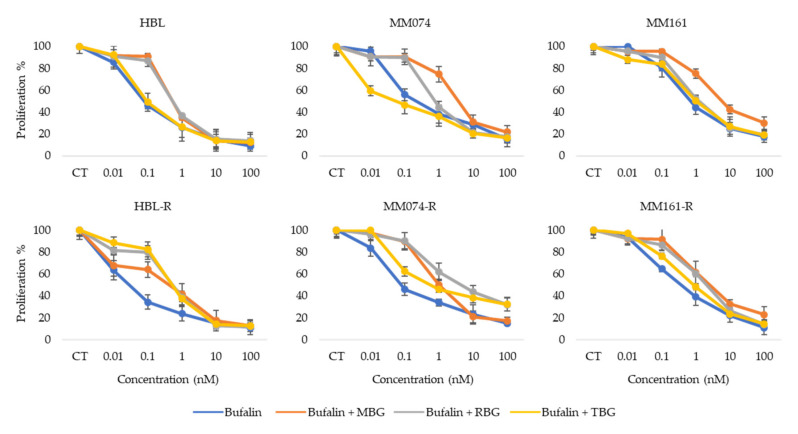
Graphs representing the percentage of viability (%) (*y* axis) at different concentrations (nM) (*x* axis) of bufalin alone or in combination with MBG, RBG or TBG in each cell line.

Finally, we wanted to see if an additive or synergic effect could appear when combining bufalin, which showed the best antiproliferative effect on the 6 melanoma cell lines, with the three other bufadienolides (Figure 5).

These data indicate that there is no additive or synergistic effect observed when combining bufalin with another bufadienolide, MBG, RBG and TBG in this case. The antiproliferative effect in the different combination tested is either similar to or weaker than the effect of bufalin alone. Thus, indicating that bufalin used alone (without any other bufadienolides) seems to be the most interesting antiproliferative molecule within fraction F1 itself obtained from the methanolic crude extract of *Rhinella marina* venom.

### 3.6. Na^+^/K^+^-ATPase Pump Expression in Sensitive and Resistant Melanoma Cell Lines

**Figure 6:**
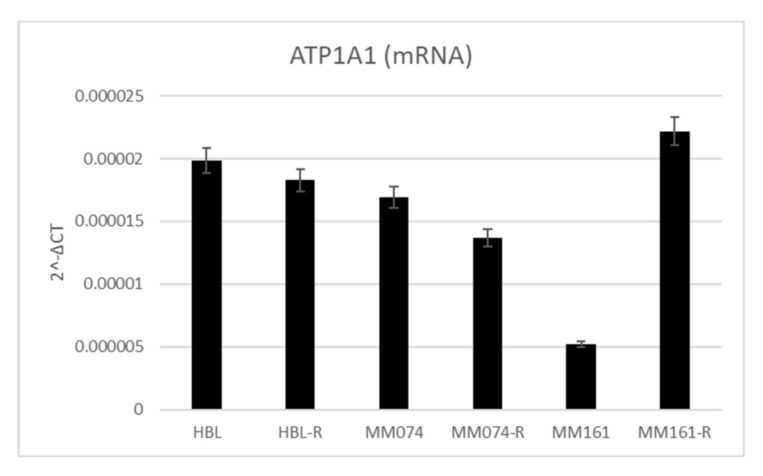
Graph representing the relative expression (2^-ΔCT) of ATP1A1 in the 6 studied melanoma cell lines.

To confirm the hypothesis that bufadienolides decrease cell viability by interacting with the sodium pump, we quantified ATP1A1 mRNA in our 6 cell lines by qPCR. The results indicated that MM161 cell line was the one with the smaller ATP1A1 mRNA content, this is also the cell line with the highest IC50 (Figure 6). This indicated that ATP1A1 mRNA content negatively correlate with bufalin efficiency in melanoma cell lines (*p* = 0.047, Pearson correlation).

## 4. Discussion

The aim of this work was to find a new potential therapeutic strategy to treat metastatic melanoma cells, either sensitive or resistant to current targeted therapies. In this context, the venoms of two toads were considered. For this study, we evaluated the antiproliferative activity of: (i) the methanolic crude extracts of the toad venoms; (ii) the fractions obtained following the fractionation process of the crude extracts; (iii) the biomolecules (alone or in combination) present in the fraction yielding the strongest antiproliferative effect.

To start off, the comparison of the antiproliferative effects of methanolic crude extracts from the venoms of *Bufo bufo* and *Rhinella marina* indicated very similar activities in the 6 studied cell lines excepted for the MM161-R cell line (NRAS mutation; resistant to pimasertib). In this cell line, the crude extract from *Rhinella marina* venom had an antiproliferative effect 10 times greater compared to the *Bufo bufo* extract. The IC_50_ for *Rhinella marina* methanolic crude extract is around 0.05 µg/mL in both sensitive and resistant cell lines (excepted for MM074-R IC_50_ = 11.50 µg/mL). Previous studies had already reported the antiproliferative activity of *Rhinella marina* crude extracts on different cancer types (gastric, bladder, lung, liver, colorectal and breast cancers), but with IC_50_ values 1.5 to 800 times higher than the ones we obtained on melanoma cells [27,36]. Its antiproliferative properties on chemo-resistant cancer cells has also been highlighted on leukemia cells harboring multidrug resistance with an IC_50_ = 0.18 µg/mL [26].

Considering the previous results on crude extracts, we decided to focus more on the *Rhinella marina* extract. After fractionation of the crude extract, we obtained four fractions: one containing mainly bufadienolides, two containing conjugated hydrophilic bufagenins, better known as bufotoxins and one for which the main compound is an indole alkaloid. The fractionation method allowed to separate compounds based on their hydrophobic properties. The compounds eluted in fraction F1 were more apolar. The fractions that were collected later in the process contained more polar compounds. Interestingly, the fraction containing the alkaloid displayed a very weak effect on viability in our 6 melanoma cell lines. This was rather unexpected considering that alkaloids are already commonly used as chemotherapeutic agents in many cancer types [37]. The data analysis indicated that fraction F1, containing mainly bufadienolides, displayed the highest antiproliferative properties. Indeed, it generates a dose-dependent response in both sensitive and resistant melanoma cell lines with IC_50_ values 8 to 60 times smaller than the ones obtained with fractions F2 and F3 containing bufotoxins. Hence, bufadienolides seem to be the class of molecules with the best anticancer activities in the tested methanolic crude extract of *Rhinella marina* venom. These bufadienolides have previously been identified in the venom of *Rhinella marina* and several studies have already highlighted the anticancer properties of bufadienolides in vitro and in vivo in many cancer types, partly by modulation of apoptosis [27,38,39,40]. A previous study indicated that bufadienolides were up to 80-fold more selective against leukemia cells when compared to dividing leukocytes. This study also indicated that bufadienolides from *Rhinella marina* did not cause hemolysis even at high concentrations, suggesting that the mechanism of cytotoxicity is probably related to a more specific pathway rather than causing direct membrane damages [32].

Bufadienolides are cardiotonic steroids that can be found in many plant or animal species, but their main sources are skin and parotid gland secretions of venomous toads [41]. They can also be found endogenously in mammals, including humans, in salt-sensitive hypertensive states such as congestive heart-failure or preeclampsia [42,43,44,45]. Bufadienolides possess a steroid structure with a δ-lactone ring at carbon C17 of ring D and they are synthetized from cholesterol as precursor through the mevalonate-independent pathways [28,46]. In mammals, they are produced in the adrenal cortex through a pathway independent of cholesterol side-chain cleavage [47]. Bufadienolides regulates the Na^+^/K^+^-ATPase pump by inhibiting the cardiotonic steroid dependent-site [15,23]. In cancer cells, the Na^+^/K^+^-ATPase pump also acts as a scaffolding protein when localized in caveolae. In this case, the pump acts as a signal transducer and regulates many processes implicated in tumor development [48,49,50]. The pump is overexpressed in many cancer types, including melanoma [24]. Therefore, one hypothesis could be that bufadienolides decrease cell viability in our in vitro models by interacting with this pump in caveolae. Our ATP1A1 mRNA quantification by qPCR indicating a negative correlation between ATP1A1 mRNA content and bufalin efficiency tend to confirm the hypothesis that bufalin acts through the Na^+^/K^+^-ATPase pump.

The chemical profile of fraction F1 was determined. Four compounds (all bufadienolides) were highlighted within the fraction of interest. Their antiproliferative activity was assessed on our 6-melanoma cell lines. The two bufadienolides with an epoxy group in C14-C15, namely MBG and RBG, had similar response patterns and similar IC_50_ s. Interestingly, in hypertensive states, MBG and RBG have opposite effects [51]. This seems to indicate that MBG and RBG have the same target on the sodium pump. Of note, their effect on the ion transport activity of the pump is opposite while it seems the effect on the signal transducer function of the pump is similar. The bufadienolides without an epoxy group in C14-C15 (bufalin and TBG) display better antiproliferative properties than the ones with the epoxy group. Among these, bufalin exhibits the best antiproliferative activity is with IC_50_ values ranging between 0.04 and 0.93 nM. Bufalin presents the same dose-dependent response pattern in both sensitive and resistant melanoma cells. This indicates that bufalin could be an interesting therapeutic strategy for patients presenting resistances to therapies targeting the MAPK pathway. More interestingly in our case, a couple of studies already investigated the potential activity of bufalin against melanoma. Although the exact mechanism of action still remains unclear, one of these studies suggested that bufalin could act by triggering apoptosis through extrinsic and mitochondria-mediated signal pathways [52]. While the other one investigated what role bufalin played in melanogenesis [53]. Their study found that bufalin promoted melanin synthesis by potentially stimulating tyrosinase activity. This could lead to the production of toxic melanin precursors which in turn could inhibit melanoma growth. Interestingly, a recent study indicated that bufalin was produced endogenously in human. LC–MS/MS analyses allowed to quantify bufalin in the serum of healthy donors at a mean concentration of 5.7 nM. This concentration dropped to 1.3 nM in patient with hepatocellular carcinoma. These data are very interesting for the potential use of bufalin in clinic. Indeed, the IC_50_ for melanoma cells is around 0.1 nM—which is approximately 50 times less than the circulating concentration in healthy individuals. Thus, bufalin could be used safely at the doses needed to suppress tumoral development [25].

Finally, we wanted to evaluate if the effect of bufalin on viability could be potentiated or decreased if combined with other bufadienolides. Our results indicated that the antiproliferative activity of bufalin mostly decreased when combined with MBG, RBG or TBG. This seems to indicate that bufalin is the bufadienolide with the strongest antiproliferative effect on melanoma cells within fraction F1. The anticancer effects of bufalin have already been highlighted in different cancer types, but at higher concentrations than observed during our current work on melanoma. Recent studies indicated that the effects of bufalin on cancer cells are mediated via modulation of apoptosis [54], necroptosis [55], cellular cycle [56], metastasis development [57], inflammation and oxidative stress [58] notably by targeting the MAPK, PI3K/AKT and NF-κB pathways and by acting on various receptors associated with tumor development such as VEGFR and EGFR [59]. However, no precise mechanism of action of bufalin on melanoma cells has been identified yet and thus it still needs to be further studied.

## 5. Conclusions

As metastatic melanoma is one of the deadliest cancer types, mainly due to a lack of effective therapies on the long term for patients who do not respond to immunotherapy, it is crucial to find new potential therapeutic candidates. Based on bio-guided fractionation for the isolation of compounds from toad venoms, this work focused on the study of antiproliferative properties of bufadienolides. We wanted to determine if they had antiproliferative effects on melanoma cells and which molecule or combination of molecules were responsible for the major antiproliferative activities.

Our results indicated strong antiproliferative capacities of toad venoms on melanoma cells. We found that these effects were mainly due to bufadienolides which are cardiotonic steroids acting on the Na^+^/K^+^-ATPase pump. This pump is overexpressed in many cancer types, including melanoma. Finally, our results indicated that bufalin, used alone without any other bufadienolides, was the compound with the highest antiproliferative activity on melanoma cells both sensitive and resistant to targeted therapy. The discovery of endogenous levels of bufalin in human serum which decrease in hepatocellular carcinoma also indicates that bufalin could be used both as a therapy and a biomarker.

## Figures and Tables

**Table 1:** Summary of the compound composition for each fraction obtained from the methanolic crude extract of the *Rhinella marina* venom.

Fraction	Compound Identification	Proportion (%)	Peak Number
**Fraction F1**	Telocinobufagin	2.48	1
	Marinobufagenin	65.50	2
	Bufalin	8.88	3
	Resibufogenin	10.27	4
**Fraction F2**	Suberoyl arginine	20.05	1
	Marinobufagin−3-pimeloyl-arginine	4.30	2
	Telocinobufotoxin	4.71	3
	Marinobufotoxin	41.06	4
	Marinobufagin−3-adipate-arginine	2.72	5
	Bufalitoxin	14.08	6
	Resibufotoxin	6.95	7
**Fraction F3**	Suberoyl arginine	47.83	1
	Marinobufagin−3-adipate-arginine	1.47	2
	Telocinobufagin−3-pimeloyl-arginine	0.84	3
	Marinobufagin−3-pimeloyl-arginine	5.43	4
	Telocinobufotoxin	7.31	5
	Marinobufotoxin	28.93	6
	Bufalitoxin	0.67	7
**Fraction F’**	Dehydrobufotenin	51.00	1
	Suberoyl arginine	30.31	2

## Supplementary Materials

The following are available online at https://www.mdpi.com/2079-7737/9/8/218/s1, Figure S1: UHPLC-UV/MS analyses of methanolic crude extract of *Rhinella marina* venom and methanolic crude extract of *Bufo bufo* venom; Table S1: Percentage of cell viability for each tested concentration and for each crude extract or fraction in HBL cells; Table S2: Percentage of cell viability for each tested concentration and for each bufadienolide alone or in combination with bufalin in HBL cells; Table S3: Percentage of cell viability for each tested concentration and for each crude extract or fraction in HBL-R cells; Table S4: Percentage of cell viability for each tested concentration and for each bufadienolide alone or in combination with bufalin in HBL-R cells; Table S5: Percentage of cell viability for each tested concentration and for each crude extract or fraction in MM074 cells; Table S6: Percentage of cell viability for each tested concentration and for each bufadienolide alone or in combination with bufalin in MM074 cells; Table S7: Percentage of cell viability for each tested concentration and for each crude extract or fraction in MM074-R cells; Table S8: Percentage of cell viability for each tested concentration and for each bufadienolide alone or in combination with bufalin in MM074-R cells; Table S9: Percentage of cell viability for each tested concentration and for each crude extract or fraction in MM161 cells; Table S10: Percentage of cell viability for each tested concentration and for each bufadienolide alone or in combination with bufalin in MM161 cells; Table S11: Percentage of cell viability for each tested concentration and for each crude extract or fraction in MM161-R cells; Table S12: Percentage of cell viability for each tested concentration and for each bufadienolide alone or in combination with bufalin in MM161-R cells.
