# Supplementary material for: Toad Venom Antiproliferative Activities on Metastatic Melanoma: Bio-Guided Fractionation and Screening of the Compounds of Two Different Venoms
Source: Biology (Basel). 2020 Aug 10;9(8):218. doi: 10.3390/biology9080218 (PMC7464305; doi:10.3390/biology9080218)
Supplement: Supplementary file 1 [file biology-09-00218-s001.pdf]

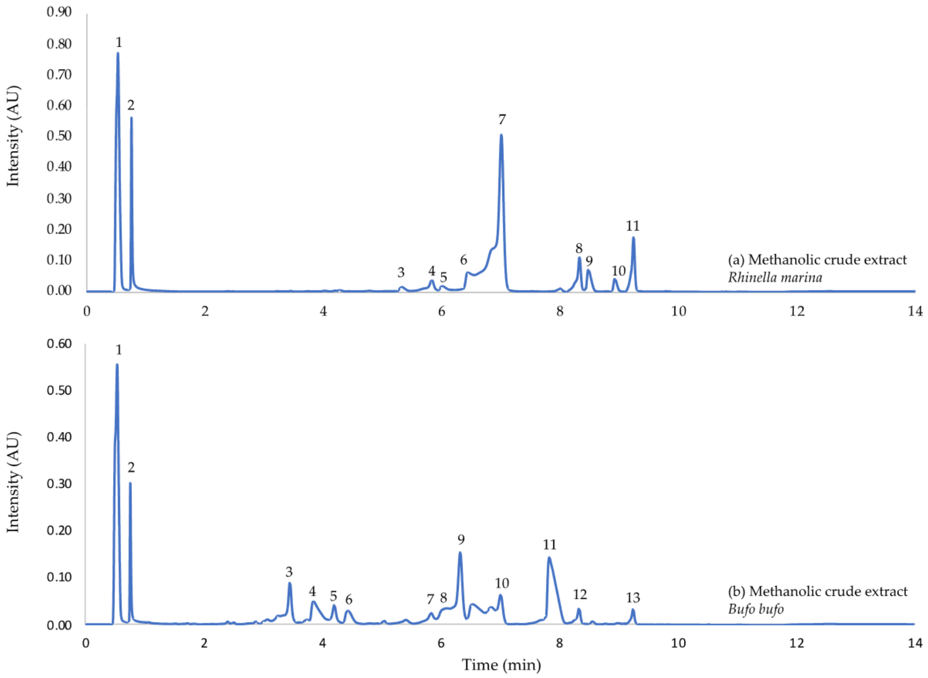


**Figure S1.** Chemical profiles of *Bufo bufo* and *Rhinella marina* crude extracts. UHPLC-UV/MS analyses of: (**a**) Methanolic crude extract of *Rhinella marina* venom – 1: dehydrobufotenin ($[M+H]^+$ = 203.2 m/z); 2: suberoyl arginine ($[M+H]^+$ = 331.2 m/z); 3: marinobufagin-3-pimeloyl-arginine ($[M+H]^+$ = 699.7 m/z); 4: telocinobufagin ($[M+H]^+$ = 403.4 m/z); 5: telocinobufotoxin ($[M+H]^+$ = 715.7 m/z); 6: marinobufotoxin ($[M+H]^+$ = 713.7 m/z); 7: marinobufagenin ($[M+H]^+$ = 401.4 m/z); 8 : bufalin ($[M+H]^+$ = 387.4 m/z); 9: bufalitoxin ($[M+H]^+$ = 699.7 m/z); 10: resibufotoxin ($[M+H]^+$ = 697.7 m/z); 11: resibufogenin ($[M+H]^+$ = 385.3 m/z). (b) Methanolic crude extract of *Bufo bufo* venom – 1: dehydrobufotenin ($[M+H]^+$ = 203.2 m/z); 2: suberoyl arginine ($[M+H]^+$ = 331.2 m/z); 3: arenobufagenin ($[M+H]^+$ = 417.4 m/z); 4: gamabufotalin-3-pelargonate-arginine ($[M+H]^+$ = 729.7 m/z); 5: unknown identity; 6: bufalin-3-sebacate-arginine ($[M+H]^+$ = 727.6 m/z); 7: telocinobufagin ($[M+H]^+$ = 403.4 m/z); 8: telocinobufotoxin ($[M+H]^+$ = 715.7 m/z); 9: bufotalin ($[M+H]^+$ = 445.4 m/z); 10: marinobufagenin ($[M+H]^+$ = 401.4 m/z); 11: 3-oxo-resibufogenin ($[M+H]^+$ = 757.7 m/z); 12: bufalin ($[M+H]^+$ = 387.3 m/z); 13: resibufogenin ($[M+H]^+$ = 385.3 m/z).

**Table S1.** Percentage of cell viability for each tested concentration and for each crude extract or fraction in HBL cells.

| HBL | 0.001 µg/mL | 0.01 µg/mL | 0.1 µg/mL | 1 µg/mL | 10 µg/mL |
|---|---|---|---|---|---|
| **Bufo bufo** | 95.02 ± 5.95 * | 74.09 ± 5.25 * | 34.92 ± 1.70 ** | 11.84 ± 0.89 *** | 7.57 ± 0.32 *** |
| **Rhinella marina** | 91.89 ± 4.05 * | 87.72 ± 6.93 * | 35.95 ± 8.29 * | 12.12 ± 0.67 *** | 5.81 ± 0.32 *** |
| **Fraction 1** | 99.41 ± 4.55 | 90.76 ± 1.92 *** | 42.29 ± 4.11 *** | 17.62 ± 3.16 *** | 11.25 ± 1.67 *** |
| **Fraction 2** | 99.58 ± 3.33 * | 99.14 ± 2.76 * | 91.33 ± 4.69 ** | 31.04 ± 2.91 *** | 14.08 ± 3.12 *** |
| **Fraction 3** | 99.73 ± 1.96 | 95.85 ± 3.66 * | 96.51 ± 3.64 * | 88.85 ± 1.69 *** | 5.27 ± 3.07 *** |
| **Fraction F′** | 99.42 ± 3.52 | 99.15 ± 2.39 | 99.18 ± 2.19 | 99.31 ± 2.96 | 91.08 ± 4.76 * |
| **Internal Mix** | 95.90 ± 3.76 *** | 38.53 ± 2.68 *** | 16.28 ± 5.70 *** | 13.97 ± 5.74 *** | 13.97 ± 3.75 *** |

Data are presented as mean ± standard deviation. Statistical significance is resulting from Student t tests and is presented as follows: * $p < 0.05$, ** $p < 0.01$, *** $p < 0.001$.

**Table S2.** Percentage of cell viability for each tested concentration and for each bufadienolide alone or in combination with bufalin in HBL cells.

| HBL | 0.00001 µM | 0.0001 µM | 0.001 µM | 0.01 µM | 0.1 µM | 1 µM | 10 µM |
|---|---|---|---|---|---|---|---|
| **MBG** | 99.92 ± 6.31 | 99.61 ± 5.52 | 99.99 ± 4.35 | 99.37 ± 3.40 | 31.11 ± 2.90 *** | 13.73 ± 5.73 *** | 10.76 ± 5.89 *** |
| **RBG** | 99.97 ± 7.12 | 98.91 ± 5.67 | 95.71 ± 6.66 | 82.64 ± 5.43 *** | 28.05 ± 3.33 *** | 18.90 ± 4.67 *** | 19.78 ± 4.67 *** |
| **Bufalin** | 85.32 ± 1.86 *** | 46.05 ± 5.10 *** | 26.15 ± 7.49 *** | 19.67 ± 5.60 *** | 14.12 ± 8.46 *** | 11.26 ± 5.76 *** | 11.87 ± 5.09 *** |
| **TBG** | 99.89 ± 2.65 | 99.51 ± 5.23 | 81.84 ± 6.06 *** | 23.84 ± 4.30 *** | 17.20 ± 4.27 *** | 14.66 ± 5.73 *** | 13.77 ± 4.73 *** |
| **Bufalin + MBG** | 91.24 ± 5.44 * | 93.92 ± 2.27 * | 34.96 ± 9.16 *** | 11.83 ± 7.40 *** | 10.10 ± 6.04 *** | / | / |
| **Bufalin + RBG** | 90.68 ± 2.83 *** | 86.75 ± 2.28 *** | 36.70 ± 5.63 *** | 7.34 ± 3.63 *** | 3.99 ± 1.82 *** | / | / |
| **Bufalin + TBG** | 92.32 ± 4.31 ** | 49.17 ± 8.27 *** | 16.29 ± 9.36 *** | 7.01 ± 3.96 *** | 4.76 ± 1.82 *** | / | / |

Data are presented as mean ± standard deviation. Statistical significance is resulting from Student t tests and is presented as follows: * $p < 0.05$, ** $p < 0.01$, *** $p < 0.001$.

**Table S3.** Percentage of cell viability for each tested concentration and for each crude extract or fraction in HBL-R cells.

| HBL-R | 0.001 µM | 0.01 µM | 0.1 µM | 1 µM | 10 µM |
|---|---|---|---|---|---|
| **Bufo bufo** | 98.03 ± 4.87 * | 66.57 ± 4.76 ** | 29.61 ± 1.22 *** | 18.69 ± 0.98 *** | 16.18 ± 0.91 *** |
| **Rhinella marina** | 99.79 ± 3.06 | 66.57 ± 3.78 | 38.22 ± 2.07 ** | 18.21 ± 0.66 *** | 14.66 ± 0.52 *** |
| **Fraction 1** | 99.74 ± 2.79 * | 99.76 ± 2.66* | 34.06 ± 2.87 *** | 11.63 ± 2.67 *** | 6.20 ± 1.80 *** |
| **Fraction 2** | 99.16 ± 1.63 | 99.94 ± 2.40 | 85.38 ± 4.60 *** | 26.15 ± 5.80 *** | 7.48 ± 5.40 *** |
| **Fraction 3** | 99.73 ± 1.96 | 95.85 ± 3.66 | 96.51 ± 3.64 | 88.58 ± 1.69 | 5.27 ± 3.07 *** |
| **Fraction F′** | 99.05 ± 2.79 | 99.27 ± 2.66 | 99.73 ± 2.87 | 99.06 ± 2.67 | 98.67 ± 1.80 |
| **Internal Mix** | 82.80 ± 2.68 *** | 37.00 ± 8.07 *** | 23.48 ± 4.97 *** | 16.11 ± 5.97 *** | 11.14 ± 3.46 *** |

Data are presented as mean ± standard deviation. Statistical significance is resulting from Student t tests and is presented as follows: * $p < 0.05$, ** $p < 0.01$, *** $p < 0.001$.

**Table S4.** Percentage of cell viability for each tested concentration and for each bufadienolide alone or in combination with bufalin in HBL-R cells.

| HBL-R | 0.00001 µM | 0.0001 µM | 0.001 µM | 0.01 µM | 0.1 µM | 1 µM | 10 µM |
|---|---|---|---|---|---|---|---|
| **MBG** | 99.41 ± 3.17 | 99.47 ± 4.80 | 93.79 ± 7.05 | 80.07 ± 6.93 *** | 34.22 ± 6.21 *** | 19.89 ± 1.91 *** | 17.76 ± 7.79 *** |
| **RBG** | 99.59 ± 2.63 | 99.52 ± 9.22 | 97.88 ± 4.58 * | 73.86 ± 3.19 *** | 37.70 ± 9.45 *** | 31.80 ± 7.73 *** | 30.59 ± 9.19 *** |
| **Bufalin** | 63.41 ± 1.69 *** | 34.28 ± 2.91 *** | 23.67 ± 4.46 *** | 18.08 ± 6.22 *** | 12.82 ± 3.89 *** | 12.93 ± 7.99 *** | 10.85 ± 7.84 *** |
| **TBG** | 99.63 ± 4.20 | 95.42 ± 4.74 | 53.64 ± 4.11 *** | 28.45 ± 5.40 *** | 19.90 ± 10.46 *** | 18.41 ± 7.89 *** | 16.69 ± 8.40 *** |
| **Bufalin + MBG** | 67.61 ± 9.72 *** | 66.90 ± 7.24 *** | 41.47 ± 9.72 *** | 17.33 ± 9.51 *** | 9.72 ± 5.35 *** | / | / |
| **Bufalin + RBG** | 82.28 ± 3.13 *** | 79.48 ± 2.46 *** | 38.72 ± 4.53 *** | 12.68 ± 6.55 *** | 6.79 ± 3.69 *** | / | / |
| **Bufalin + TBG** | 93.55 ± 6.94 | 92.37 ± 3.25 | 37.42 ± 3.00 *** | 12.05 ± 5.88 *** | 7.51 ± 3.80 *** | / | / |

Data are presented as mean ± standard deviation. Statistical significance is resulting from Student t tests and is presented as follows: * $p < 0.05$, *** $p < 0.001$.

**Table S5.** Percentage of cell viability for each tested concentration and for each crude extract or fraction in MM074 cells.

| MM074 | 0.001 µM | 0.01 µM | 0.1 µM | 1 µM | 10 µM |
|---|---|---|---|---|---|
| **Bufo bufo** | 98.79 ± 3.74 | 96.16 ± 4.88 | 40.51 ± 2.10 ** | 25.97 ± 1.36 ** | 23.64 ± 1.21 ** |
| **Rhinella marina** | 97.12 ± 6.71 | 95.06 ± 5.30 | 45.49 ± 3.26 ** | 24.28 ± 1.85 ** | 21.11 ± 1.69 ** |
| **Fraction 1** | 99.56 ± 4.47 | 93.16 ± 3.19 ** | 44.63 ± 4.61 *** | 18.18 ± 6.03 *** | 13.58 ± 3.22 *** |
| **Fraction 2** | 92.27 ± 4.62 *** | 91.92 ± 4.18 *** | 90.59 ± 4.22 *** | 28.44 ± 4.86 *** | 16.41 ± 3.96 *** |
| **Fraction 3** | 96.76 ± 4.77 * | 94.98 ± 3.82 * | 93.31 ± 2.22 *** | 81.59 ± 5.14 *** | 6.32 ± 2.72 *** |

| | | | | | |
|---|---|---|---|---|---|
| **Fraction F′** | 94.90 ± 2.24 ** | 91.98 ± 3.95 ** | 90.60 ± 3.40 *** | 86.48 ± 3.26 *** | 83.87 ± 3.60 *** |
| **Internal Mix** | 93.81 ± 9.75 | 57.10 ± 8.49 *** | 35.08 ± 6.99 *** | 33.67 ± 8.01 *** | 31.62 ± 8.64 *** |

Data are presented as mean ± standard deviation. Statistical significance is resulting from Student t tests and is presented as follows: * $p < 0.05$, ** $p < 0.01$, *** $p < 0.001$.

**Table S6.** Percentage of cell viability for each tested concentration and for each bufadienolide alone or in combination with bufalin in MM074 cells.

| MM074 | 0.00001 μM | 0.0001 μM | 0.001 μM | 0.01 μM | 0.1 μM | 1 μM | 10 μM |
|---|---|---|---|---|---|---|---|
| **MBG** | 96.36 ± 4.35 | 95.43 ± 4.29 | 97.44 ± 1.73 | 95.54 ± 2.08 * | 52.37 ± 1.24 *** | 27.17 ± 4.34 *** | 25.06 ± 5.76 *** |
| **RBG** | 99.34 ± 6.71 | 95.68 ± 4.12 | 98.83 ± 4.55 | 94.95 ± 3.77 * | 55.89 ± 5.15 *** | 34.00 ± 4.39 *** | 30.74 ± 3.66 *** |
| **Bufalin** | 95.57 ± 4.97 * | 55.88 ± 5.75 *** | 38.05 ± 3.74 *** | 30.43 ± 4.99 *** | 27.19 ± 3.80 *** | 25.98 ± 7.86 *** | 28.11 ± 7.33 *** |
| **TBG** | 98.23 ± 9.36 | 99.67 ± 7.89 | 99.68 ± 6.70 * | 55.40 ± 4.73 *** | 37.79 ± 6.68 *** | 36.56 ± 4.59 *** | 37.84 ± 2.01 *** |
| **Bufalin + MBG** | 92.64 ± 9.28 * | 90.16 ± 8.27 * | 74.54 ± 7.08 *** | 31.07 ± 6.91 *** | 21.85 ± 6.00 *** | / | / |
| **Bufalin + RBG** | 90.47 ± 3.07 | 89.63 ± 8.71 | 44.20 ± 4.14 *** | 21.42 ± 5.62 *** | 17.56 ± 3.12 *** | / | / |
| **Bufalin + TBG** | 59.38 ± 4.37 *** | 46.41 ± 7.78 *** | 36.17 ± 9.30 *** | 20.44 ± 3.87 *** | 16.53 ± 3.35 *** | / | / |

Data are presented as mean ± standard deviation. Statistical significance is resulting from Student t tests and is presented as follows: * $p < 0.05$, *** $p < 0.001$.

**Table S7.** Percentage of cell viability for each tested concentration and for each crude extract or fraction in MM074-R cells.

| MM074-R | 0.001 μM | 0.01 μM | 0.1 μM | 1 μM | 10 μM |
|---|---|---|---|---|---|
| **Bufo bufo** | 98.15 ± 4.74 | 71.56 ± 3.65 | 64.06 ± 2.76 ** | 56.53 ± 1.10 ** | 52.31 ± 1.68 ** |
| **Rhinella marina** | 99.54 ± 4.06 | 96.57 ± 4.95 | 67.51 ± 2.79 ** | 58.16 ± 1.89 *** | 54.77 ± 2.06 ** |
| **Fraction 1** | 93.53 ± 3.86 * | 81.03 ± 4.01 *** | 34.01 ± 3.97 *** | 16.73 ± 4.87 *** | 11.86 ± 3.94 *** |
| **Fraction 2** | 99.86 ± 1.08 | 99.91 ± 2.12 | 83.48 ± 2.56 *** | 36.72 ± 2.79 *** | 22.31 ± 2.26 *** |
| **Fraction 3** | 99.55 ± 3.71 | 96.15 ± 4.25 * | 94.80 ± 2.63 *** | 71.80 ± 4.11 *** | 5.13 ± 2.45 *** |
| **Fraction F′** | 99.95 ± 5.10 | 98.37 ± 5.16 | 94.25 ± 6.74 * | 92.56 ± 6.76 * | 77.44 ± 7.58 *** |
| **Internal Mix** | 99.54 ± 8.03 | 56.73 ± 3.00 *** | 36.90 ± 4.09 *** | 30.18 ± 5.46 *** | 30.39 ± 3.83 *** |

Data are presented as mean ± standard deviation. Statistical significance is resulting from Student t tests and is presented as follows: * $p < 0.05$, ** $p < 0.01$, *** $p < 0.001$.

**Table S8.** Percentage of cell viability for each tested concentration and for each bufadienolide alone or in combination with bufalin in MM074-R cells.

| MM074-R | 0.00001 μM | 0.0001 μM | 0.001 μM | 0.01 μM | 0.1 μM | 1 μM | 10 μM |
|---|---|---|---|---|---|---|---|
| **MBG** | 99.54 ± 6.21 | 99.55 ± 10.37 | 99.13 ± 8.47 * | 95.75 ± 6.75 | 41.72 ± 3.36 *** | 26.01 ± 2.99 *** | 24.8 ± 2.13 *** |
| **RBG** | 92.27 ± 6.52 | 99.54 ± 4.29 | 96.91 ± 2.25 | 92.66 ± 2.46 * | 47.95 ± 8.50 *** | 37.15 ± 4.09 *** | 42.48 ± 4.78 *** |
| **Bufalin** | 83.55 ± 1.95 *** | 45.94 ± 3.27 *** | 36.93 ± 4.60 *** | 29.11 ± 2.74 *** | 65.80 ± 2.74 *** | 26.32 ± 3.11 *** | 26.18 ± 5.72 *** |
| **TBG** | 99.72 ± 5.43 | 99.88 ± 4.77 | 78.85 ± 5.41 *** | 42.77 ± 4.93 *** | 30.02 ± 3.40 *** | 27.87 ± 4.86 *** | 26.85 ± 3.47 *** |
| **Bufalin + MBG** | 97.32 ± 5.45 * | 89.59 ± 7.31 * | 49.81 ± 8.15 *** | 20.76 ± 6.07 *** | 17.31 ± 5.75 *** | / | / |
| **Bufalin + RBG** | 99.94 ± 7.97 | 99.05 ± 5.05 | 61.84 ± 5.45 *** | 43.52 ± 3.39 *** | 32.02 ± 9.56 *** | / | / |
| **Bufalin + TBG** | 99.77 ± 6.82 | 62.23 ± 9.56 *** | 45.93 ± 4.09 *** | 38.11 ± 2.70 *** | 32.34 ± 4.55 *** | / | / |

Data are presented as mean ± standard deviation. Statistical significance is resulting from Student t tests and is presented as follows: * $p < 0.05$, *** $p < 0.001$.

**Table S9.** Percentage of cell viability for each tested concentration and for each crude extract or fraction in MM161 cells.

| MM161 | 0.001 µM | 0.01 µM | 0.1 µM | 1 µM | 10 µM |
|---|---|---|---|---|---|
| Bufo bufo | 99.07 ± 3.99 | 98.01 ± 2.90 | 60.98 ± 2.51 ** | 38.11 ± 4.31 ** | 29.14 ± 1.99 ** |
| Rhinella marina | 92.71 ± 3.18 | 96.57 ± 4.65 | 63.45 ± 2.85 ** | 33.88 ± 1.62 ** | 26.89 ± 2.27 ** |
| Fraction 1 | 95.66 ± 2.39 * | 96.47 ± 3.54 | 70.91 ± 4.37 *** | 30.23 ± 5.02 *** | 15.00 ± 5.39 *** |
| Fraction 2 | 99.25 ± 4.29 | 99.49 ± 3.51 | 94.60 ± 3.42 | 54.11 ± 5.47 *** | 18.71 ± 4.62 *** |
| Fraction 3 | 99.65 ± 4.91 | 96.79 ± 3.41 * | 95.26 ± 4.31 *** | 85.20 ± 6.72 *** | 6.18 ± 2.74 *** |
| Fraction F′ | 99.24 ± 3.04 | 99.62 ± 1.55 | 97.60 ± 1.58 * | 96.69 ± 1.12 *** | 91.16 ± 2.93 *** |
| Internal Mix | 99.13 ± 5.41 | 67.42 ± 3.73 *** | 33.93 ± 4.92 *** | 20.86 ± 5.87 *** | 11.92 ± 2.59 *** |

Data are presented as mean ± standard deviation. Statistical significance is resulting from Student t tests and is presented as follows: * $p < 0.05$, ** $p < 0.01$, *** $p < 0.001$.

**Table S10.** Percentage of cell viability for each tested concentration and for each bufadienolide alone or in combination with bufalin in MM161 cells.

| MM161 | 0.00001 µM | 0.0001 µM | 0.001 µM | 0.01 µM | 0.1 µM | 1 µM | 10 µM |
|---|---|---|---|---|---|---|---|
| MBG | 99.33 ± 5.62 | 98.20 ± 5.09 | 99.85 ± 7.36 | 99.33 ± 2.98 | 68.51 ± 6.43 *** | 25.72 ± 6.43 *** | 15.06 ± 4.84 *** |
| RBG | 99.20 ± 5.38 | 99.20 ± 5.84 | 95.99 ± 3.74 * | 95.67 ± 3.63 * | 60.68 ± 4.81 *** | 33.83 ± 6.91 *** | 25.08 ± 5.87 *** |
| Bufalin | 99.81 ± 3.97 | 80.5 ± 4.26 *** | 44.14 ± 5.67 *** | 28.98 ± 4.70 *** | 20.57 ± 5.67 *** | 15.39 ± 6.00 *** | 13.22 ± 3.82 *** |
| TBG | 99.88 ± 2.59 | 98.69 ± 1.09 | 93.12 ± 1.59 * | 48.15 ± 6.14 *** | 29.35 ± 6.95 *** | 21.20 ± 9.41 *** | 20.23 ± 9.57 *** |
| Bufalin + MBG | 95.41 ± 4.22 * | 95.27 ± 3.98 * | 75.21 ± 5.66 *** | 42.30 ± 5.44 *** | 30.01 ± 9.99 *** | / | / |
| Bufalin + RBG | 95.50 ± 5.76 | 89.91 ± 4.86 ** | 52.22 ± 8.78 *** | 25.52 ± 4.52 *** | 19.25 ± 7.12 *** | / | / |
| Bufalin + TBG | 88.07 ± 7.41 | 83.63 ± 7.37 *** | 50.25 ± 2.96 *** | 27.07 ± 3.14 *** | 18.92 ± 7.62 *** | / | / |

Data are presented as mean ± standard deviation. Statistical significance is resulting from Student t tests and is presented as follows: * $p < 0.05$, ** $p < 0.01$, *** $p < 0.001$.

**Table S11.** Percentage of cell viability for each tested concentration and for each crude extract or fraction in MM161-R cells.

| MM161-R | 0.001 µM | 0.01 µM | 0.1 µM | 1 µM | 10 µM |
|---|---|---|---|---|---|
| Bufo bufo | 84.05 ± 3.19 *** | 63.64 ± 7.39 *** | 37.05 ± 7.65 *** | 32.99 ± 7.44 *** | 19.55 ± 3.05 *** |
| Rhinella marina | 96.85 ± 5.38 | 88.33 ± 4.81 ** | 55.48 ± 2.09 *** | 42.40 ± 2.61 *** | 32.13 ± 3.42 *** |
| Fraction 1 | 97.02 ± 3.91 | 84.50 ± 2.90 *** | 33.79 ± 4.45 *** | 15.97 ± 5.90 *** | 11.40 ± 4.38 *** |
| Fraction 2 | 91.16 ± 4.66 * | 87.28 ± 7.00 *** | 78.52 ± 6.79 *** | 27.64 ± 8.20 *** | 17.41 ± 6.87 *** |
| Fraction 3 | 99.97 ± 5.49 | 99.78 ± 5.56 * | 97.74 ± 5.44 | 62.64 ± 4.14 *** | 10.88 ± 5.42 *** |
| Fraction F′ | 99.31 ± 6.70 | 99.01 ± 5.64 | 99.73 ± 4.27 | 98.99 ± 5.46 | 77.24 ± 5.99 *** |
| Internal Mix | 96.39 ± 2.37 * | 57.99 ± 4.76 *** | 34.62 ± 4.68 *** | 25.81 ± 8.92 *** | 16.01 ± 7.97 *** |

Data are presented as mean ± standard deviation. Statistical significance is resulting from Student t tests and is presented as follows: * $p < 0.05$, ** $p < 0.01$, *** $p < 0.001$.

**Table S12.** Percentage of cell viability for each tested concentration and for each bufadienolide alone or in combination with bufalin in MM161-R cells.

| MM161-R | 0.00001 µM | 0.0001 µM | 0.001 µM | 0.01 µM | 0.1 µM | 1 µM | 10 µM |
|---|---|---|---|---|---|---|---|
| MBG | 96.62 ± 7.43 | 97.43 ± 2.45 | 89.76 ± 3.63 * | 90.35 ± 3.43 * | 49.22 ± 5.27 *** | 25.08 ± 6.40 *** | 17.28 ± 6.38 *** |
| RBG | 97.64 ± 2.48 | 94.92 ± 4.75 | 96.42 ± 6.66 | 88.15 ± 3.74 ** | 50.49 ± 8.87 *** | 37.45 ± 8.56 *** | 30.83 ± 8.56 *** |
| Bufalin | 93.32 ± 3.34 | 64.62 ± 2.59 *** | 39.01 ± 7.55 *** | 27.06 ± 6.40 *** | 18.06 ± 6.26 *** | 14.96 ± 8.25 *** | 13.98 ± 7.42 *** |
| TBG | 99.30 ± 4.13 | 95.05 ± 5.31 | 88.11 ± 2.14 *** | 42.78 ± 3.31 *** | 25.56 ± 10.08 *** | 19.69 ± 6.12 *** | 17.98 ± 7.34 *** |

| | | | | | | | |
|---|---|---|---|---|---|---|---|
| **Bufalin + MBG** | 92.17 ± 4.74 | 95.65 ± 10.56 | 61.45 ± 10.15 *** | 32.56 ± 3.65 *** | 22.82 ± 7.31 *** | / | / |
| **Bufalin + RBG** | 92.46 ± 2.67 | 86.55 ± 2.88 * | 60.18 ± 3.71 *** | 26.46 ± 6.06 *** | 13.70 ± 8.03 *** | / | / |
| **Bufalin + TBG** | 96.98 ± 2.89 | 76.26 ± 6.39 *** | 48.05 ± 4.91 *** | 23.43 ± 4.91 *** | 14.45 ± 5.77 *** | / | / |

Data are presented as mean ± standard deviation. Statistical significance is resulting from Student t tests and is presented as follows: * $p < 0.05$, ** $p < 0.01$, *** $p < 0.001$.
